# IDEAL‐Acu: A Methodological Framework for Evaluating the Effects of Acupuncture

**DOI:** 10.1111/jebm.70043

**Published:** 2025-06-09

**Authors:** Jiali Liu, Xiaochao Luo, Yemeng Chen, Ling Zhao, Minghong Yao, Jiajie Yu, Jiahui Yang, Ling Li, Xin Sun

**Affiliations:** ^1^ Institute of Integrated Traditional Chinese and Western Medicine and Chinese Evidence‐Based Medicine Center Cochrane China Center and IDEAL China Center West China Hospital Sichuan University Chengdu China; ^2^ NMPA Key Laboratory for Real World Data Research and Evaluation in Hainan Chengdu China; ^3^ Sichuan Center of Technology Innovation for Real World Data Chengdu China; ^4^ New York College of Traditional Chinese Medicine Mineola New York NewYork; ^5^ Acupuncture and Tuina School Chengdu University of Traditional Chinese Medicine Chengdu China; ^6^ College of Acupuncture and Massage Jiangxi University of Chinese Medicine Nanchang China

**Keywords:** acupuncture, evaluation, methodological framework, recommendations

## Abstract

**Objectives:**

The demand for high‐quality clinical evidence supporting acupuncture remains urgent, necessitating the establishment of a suitable methodological framework to promote its generation.

**Methods:**

Following internal deliberations and extensive online discussions with experts in the IDEAL Collaboration, we proposed the IDEAL‐Acu framework specifically for acupuncture, based on the surgery‐focused IDEAL model with necessary modifications to accommodate the characteristics of acupuncture. To ensure consensus on recommendations, a panel of external experts and internal research team members was convened, and any disagreements were iteratively resolved through expert review.

**Results:**

This article introduces an IDEAL‐Acu framework with five stages for evaluating acupuncture outcome and improving practice to optimize treatment. The framework includes Idea (proposal of an acupuncture regime), Development (optimization or standardization of the acupuncture regime), Exploration (feasibility assessment for conducting a definitive RCT), Assessment (evaluation of effects through comparison with standard therapy or sham acupuncture), and Long‐term monitoring (examination of long‐term efficacy and safety) stages. We provide clear recommendations for each stage along with specific examples.

**Conclusion:**

The framework highlights the importance of conducting studies at each stage in acupuncture evaluation process and can serve as a helpful guide for assessing its effects and promoting evidence‐based practice in acupuncture.

## 1 Introduction

Acupuncture, a therapeutic technique in which thin needles are inserted and manipulated at specific points on the body to achieve therapeutic purpose, has been practiced in China for thousands of years [1]. It is an important component of Traditional Chinese Medicine (TCM). In recent decades, due to the side effects of pharmacological interventions, an increasing number of people have turned to acupuncture for treating disorders, maintaining wellness and improving mental health [2]. In the United States, insurance coverage for acupuncture increased from 1.0% in 2002 to 2.2% in 2022, while its use for pain management rose from approximately 55% in 2002 to around 72% in 2022 [3]. Furthermore, acupuncture has gained popularity across 183 countries and regions worldwide [4], and according to the World Health Organization (WHO) 2019 global report, 113 Member States acknowledge its use in healthcare [5].

With the widespread application of acupuncture, numerous studies have been conducted to assess its effects. However, the results remain controversial and the scientific evidence for its efficacy is mixed [6, 7]. The Acupuncture Evidence Project by the Australian Acupuncture and Chinese Medicine Association Ltd (AACMA) reviewed the effect of acupuncture for 122 conditions across 14 broad clinical areas, and found insufficient or unclear evidence for 71 (58.2%) conditions, and no evidence for five (4.1%) conditions [8]. This also reflects the fact that many acupuncture therapies are widely used to treat certain conditions before robust evidence of their clinical superiority over standard treatment or placebo/sham acupuncture is generated. There is still an urgent demand for high‐quality clinical evidence that supports the use of acupuncture.

The evaluation of acupuncture presents unique methodological and practical challenges due to its complex nature. Firstly, acupuncture treatment regimens vary greatly due to differences in acupuncture point prescriptions, manipulation techniques and dosage (i.e., number of needles, intensity, repetition intervals, needle retention time, treatment frequency and duration) [9]. This wide heterogeneity makes it challenging to establish a standardized acupuncture treatment regimen that can be consistently applied across different clinical trials, posing difficulties in replicating studies and generalizing results to other populations or settings. Secondly, the effectiveness of acupuncture treatment heavily relies on the expertise and proficiency of acupuncturists, which can vary significantly among practitioners and impact the estimation of treatment effects [10]. It is necessary to standardize operation process and acupuncturist techniques through a normative framework to achieve accuracy or consistency of acupuncture intervention, while still allowing some techniques technical flexibility. Thirdly, acupuncture treatment involves patient‐practitioner communication, which may influence its effectiveness due to positive expectancy [11]. The construction of appropriate communication methods is also important in the evaluation of acupuncture effect.

While continued efforts have been made to address the above challenges [12, 13], skepticism regarding the methodological quality of acupuncture clinical studies persists due to issues related to the lack of standardized interventions [9, 14]. The complexity of acupuncture makes it impossible to simply follow the methodological framework for evaluating pharmacological therapy. It is necessary to establish a methodological framework suitable for acupuncture, across all stages of its development, from the proposal of acupuncture treatment regimen to the long‐term results, to promote the generation of high‐quality acupuncture clinical evidence.

The IDEAL (Idea, Development, Exploration, Assessment, Long term study) framework is a model for integrated stepwise evaluation of surgery and other invasive therapies, aimed at improving the quality of research in surgery [15]. The IDEAL framework is proposed by the IDEAL Collaboration to describe what types of studies and reporting should be used for five stages of development of surgical innovations [16]. The initial stage 1 (idea) focuses on the first use of a new procedure or device in patients. The stage 2a (development) involves refining the technique, often iteratively, to achieve a final stable version. In the stage 2b (exploration) other surgeons and centers become involved and achieve consensus on technical details, indications, operator learning curves and quality control. In the stage 3 (assessment) a comparative evaluation occurs, usually against current standard therapy. Stage 4 (long‐term study) is need to recognizing late or rare events. In the 2019 update, a Pre‐IDEAL stage describing preclinical studies has been added to ensure the safety and efficacy of new interventions before they enter progress to human trials [17].

Generating high‐quality evidence for acupuncture necessitates a systematic, stepwise evaluation framework. While several methodological frameworks exist for complex interventions, the IDEAL framework stands out as particularly adaptable to acupuncture research. Other frameworks, such as the Medical Research Council (MRC) framework for complex interventions [18], focus on phased development. However, they do not offer the same explicit emphasis on iterative refinement and real‐world adaptation that the IDEAL framework provides. The strength of IDEAL framework lies in its structured yet flexible approach to innovation evaluation, which has already been successfully applied to various practitioner‐based complex therapies, such as the IDEAL‐D for the systematic evaluation of therapeutic medical devices [19], the R‐IDEAL for evaluation of radiotherapy innovations [20], and the IDEAL‐Physio for physical interventions [21]. The IDEAL framework demonstrates substantial applicability to acupuncture research, particularly in its core components such as innovation identification, exploratory stage studies, and randomized controlled trial evaluation. However, acupuncture, as a minimally invasive therapy, differs from surgical interventions in its underlying mechanisms and clinical application. Unlike surgery, acupuncture primarily operates through acupoint stimulation and the regulation of energy flow (Qi), representing a lower‐risk intervention with greater flexibility in treatment individualization [22]. These unique attributes underscore the necessity for modifications to the IDEAL framework to ensure its alignment with acupuncture research requirements. In response to this need, our research team has initiated efforts since 2018 to adapt the IDEAL framework to the unique characteristics of acupuncture [23].

## 2 Methods

The development of the IDEAL‐Acu framework followed a rigorous, iterative process involving multiple stages of internal and external expert consultations. Initially, we conducted several structured internal panel discussions among research team to draft the preliminary framework. These discussions focused on identifying key methodological challenges in acupuncture research, including its unique characteristics such as difficulty in standardization, and higher safety profile compared to surgical interventions. On July 14, 2022, we organized an online meeting with experts from the IDEAL Collaboration to comprehensively review the draft framework. We presented the initial structure, including the proposed five‐stage division, the purpose of each stage, recommended study designs, and key output requirements. Experts provided feedback on the framework's alignment with acupuncture unique characteristics and clinical research applicability. Disagreements were resolved through structured discussions, with consensus achieved by prioritizing evidence‐based principles and practical feasibility.

Subsequently, we invited a panel of ten external experts specializing in acupuncture, clinical epidemiology, and evidence‐based medicine to independently review the draft framework. These experts were selected based on their extensive experience in acupuncture research, methodological expertise, and contributions to evidence‐based medicine. Feedback was systematically categorized into three main areas, including methodological rigor, alignment with acupuncture unique characteristics, and practical applicability. Disagreements were resolved through an iterative revision process, and by revisiting the evidence, re‐evaluating the alignment with acupuncture unique characteristics, and conducting additional rounds of discussion until consensus was reached. For example, one point of debate was whether sham acupuncture should be set up in RCTs, because sham acupuncture may not be an inert placebo. After reviewing the evidence on the therapeutic effects of sham acupuncture and its impact on study validity, we decided to recommend sham controls as optional and recommend that it should be tested in stage 3 study to determine if it adequately simulates non‐specific effects associated with acupuncture therapy.

To ensure transparency and rigor, we documented each iteration of the framework, including the rationale for modifications and the resolution of disagreements. The final version of the framework was reviewed and approved by both the external expert panel and our internal research group, ensuring that all concerns were addressed and that the framework was both methodologically sound and practically applicable to acupuncture research.

## 3 Results

**FIGURE 1:**
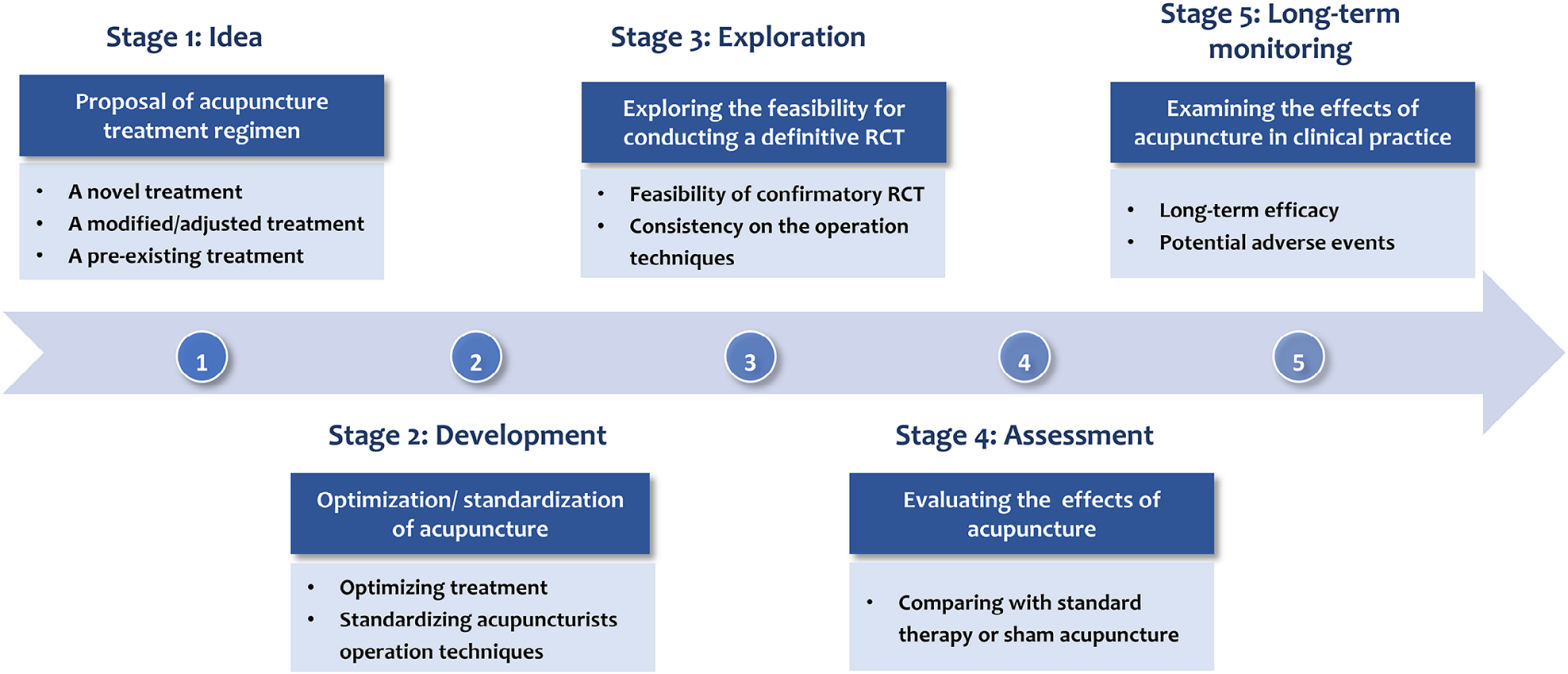
Five stages of IDEAL‐Acu framework.

**FIGURE 2:**
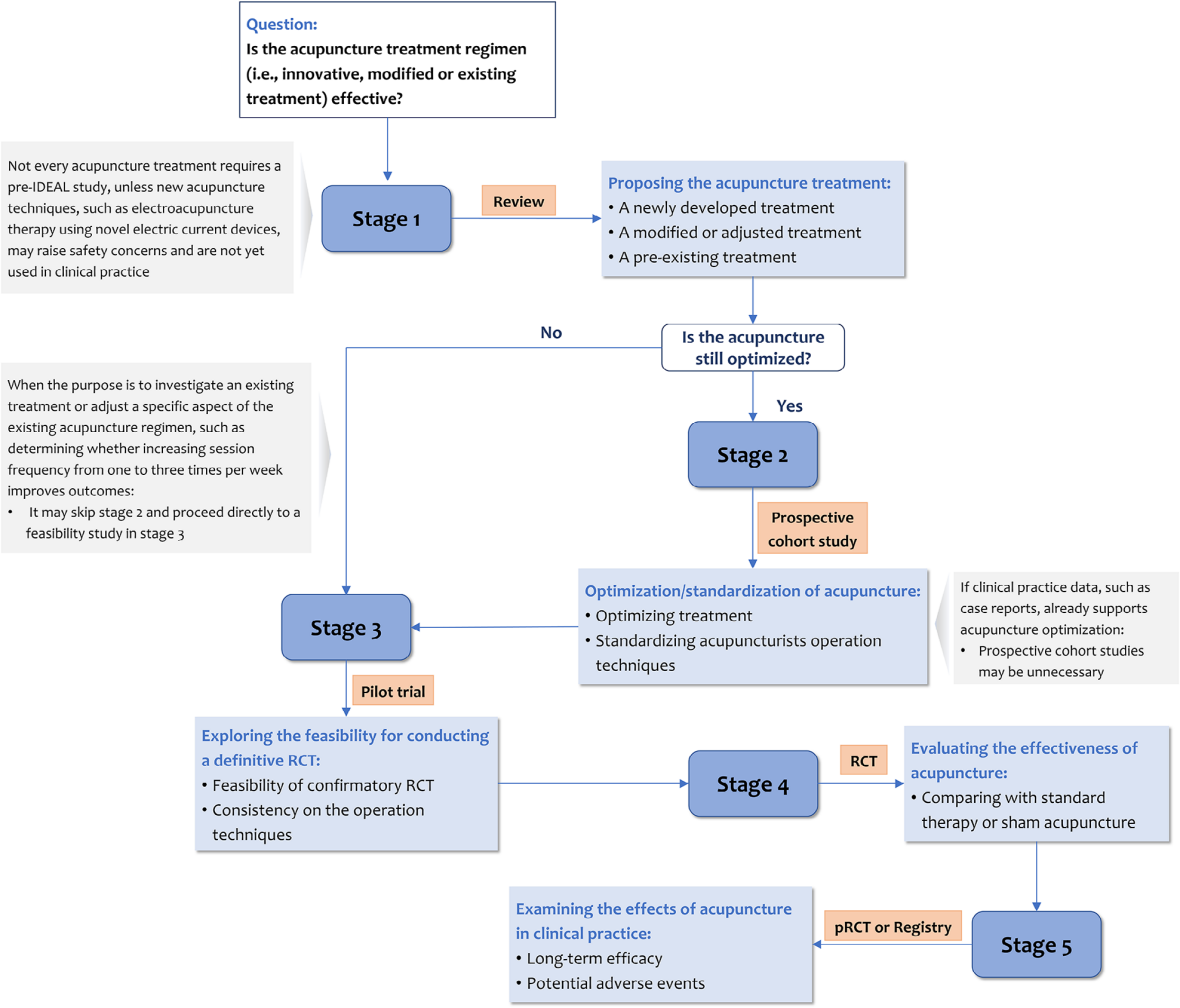
Flowchart of IDEAL‐Acu framework.

The IDEAL‐Acu framework divides acupuncture research into five stages: Idea (proposal of acupuncture regime), Development (optimization or standardization of acupuncture regime), Exploration (feasibility assessment for conducting a definitive RCT), Assessment (evaluation of effects through comparison with standard therapy or sham acupuncture), and Long‐term monitoring (examination of long‐term efficacy and safety) (Figure 1 and Table 1). The formulation of acupuncture research plans should be guided by well‐defined research questions, encompassing various stages (Figure 2).

**TABLE 1:** IDEAL‐Acu framework and recommendations.

Stage	Purpose	Study design	Key output
**Stage 1 Idea**	**Proposal of acupuncture treatment regimen** A novel treatment A modified or adjusted treatment A pre‐existing treatment	Review (e.g., ancient literature, TCM theories, renowned doctors' experience, or research evidence like case report)	Developing a preliminary acupuncture treatment regimen, including: What the acupuncture intervention is (e.g., acupoint prescription, manipulation) Target disease Potential mechanism of action, or TCM theory Possible expected effects in terms of efficacy and safety
**Stage 2 Development**	**Optimization/ standardization of acupuncture treatment regimen** Optimizing treatment Standardizing acupuncturists operation techniques	Prospective cohort study with sequential reporting of cases and modifications	An optimal acupuncture treatment regimen, including: Full details of patient characteristics Full details of optimal acupuncture intervention Possible efficacy and safety Standardized technical training programs
**Stage 3 Exploration**	**Exploring the feasibility of acupuncture regimen for conducting a definitive RCT**	Feasibility/pilot RCT	Consensus on feasibility of confirmatory RCT, including: Patient inclusion and exclusion criteria Recruitment strategies Details of control intervention Primary outcome Data collection methods Adherence to treatment protocols Estimates of expected effects Consistency on the operation techniques among acupuncturists, including: Qualification or experience level Learning curves Communication strategies
**Stage 4 Assessment**	**Evaluating the effects by comparing with current standard therapy or sham acupuncture**	RCTs (multiple centers)	Valid evidence on effects of acupuncture treatment regimen
**Stage 5 Long‐term monitoring**	**Examining the effects of acupuncture treatment regimen in clinical practice**	Pragmatic RCT or Registry	Performance of acupuncture treatment in real‐word settings, including: Long‐term effectiveness Potential adverse events

### 3.1 Pre‐IDEAL (Preclinical): Not every Acupuncture Regimen Requires Pre‐IDEAL Studies

Pre‐IDEAL study is essential prior to first in human trials of a surgical innovation. Preclinical studies can be expected to identify potential safety issues with a new device or technique for treating the targeted disease. Although most new technology innovations may follow these stages exactly, a well‐developed technology can also start at a later stage in its development or skip a certain stage. Acupuncture has been used for thousands of years, and many acupuncture techniques have been refined over generations and have lower potential risks than surgery. For example, Wang's Jiaji acupoints, a new acupoint prescription based on the experience of the famous old doctor (Le Ting Wang, 1895–1984), has been proficiently applied to the clinical practice of acupuncture [24], in which case preclinical studies such as animal experiments are not needed to determine potential safety, and clinical trials can be conducted to evaluate its efficacy/effectiveness.

The necessity of pre‐IDEAL studies in acupuncture technology innovation should depend on the specific nature of the innovation. For instance, if a new acupuncture device involves the use of electrical currents or laser, preclinical research may be necessary to ensure that the device is safe [25]. Similarly, if a new acupuncture technique involves the use of novel materials or methods [26], preclinical study may be needed. In fact, acupuncture clinical research is mainly to evaluate the acupuncture that has been applied to the clinic, rarely starts with innovative acupuncture. Existing preclinical studies of acupuncture based on animal models have focused on the mechanism of action of acupuncture in treating disease [27], rather than exploring the potential risks of a new acupuncture technique. Whether or not to conduct preclinical research in acupuncture technology innovation should be based on the specific nature of the innovation and the potential risks and benefits associated with its use. In general, not every acupuncture regimen requires pre‐IDEAL studies.

### 3.2 Stage 1 (Idea): The Proposal of Acupuncture Regimen

The idea stage answers the question what is the treatment concept and why is it needed [17]. For acupuncture, this stage may involve developing a novel treatment (e.g., a new device of electrical stimulation to acupuncture needles), modifying or adjusting an existing treatment (e.g., acupoint prescription, acupuncture manipulation, needling depths, treatment frequency, or using acupuncture in new disease areas or clinical contexts), or evaluating a pre‐existing treatment. It's important to consider the traditional principles of acupuncture and how they align with modern scientific understanding. In this stage, a preliminary acupuncture regimen should be provided.

The nature of acupuncture allows for the review of ancient literature (i.e., ancient cases), referring to traditional Chinese medicine theories, drawing upon the experience of renowned doctors, or review of research evidence (e.g., case report) to propose a preliminary acupuncture treatment regimen. This regimen should include a description of the acupuncture intervention, target disease, potential mechanism of action or TCM theory, and possible expected effects in terms of efficacy and safety.

#### 3.2.1 Example of Stage 1

According to traditional Chinese medicine theory, such as “to check if it is [a] sensitized acupoint, press it and the pain is relieved during the pressing. Here is [a] sensitized acupoint” described in the Huangdi Neijing (The Yellow Emperor's Inner Classic, ∼100 BC), these pain‐sensitized points are key indicators of disease and have significant therapeutic effects. Although acupuncture at these points is commonly used for pain diseases in clinic, there are variations in treatment regimens and uncertainty regarding its effectiveness. Drawing from traditional Chinese medicine theory and clinical experience [28], we hypothesized that acupuncture targeting highly sensitive acupoints could improve the therapeutic outcomes for primary knee osteoarthritis. We initially proposed the intervention were acupuncture at high pain‐sensitized acupoints, and acupuncture at low pain‐sensitized acupoints.

### 3.3 Stage 2 (Development): Optimization or Standardization of Acupuncture Regimen

Stage 2 (development) aims to determine whether the intervention has achieved a level of optimization or standardization that is adequate for replication by others. Developing a new surgical procedure or device requires technical proficiency and iterative testing [29]. Acupuncture also requires technical skills, but its optimization process may not be as sophisticated as surgical innovation. For acupuncture, the focus may lie in improving or standardizing the regimen, such as refining acupoint prescription, optimizing needling techniques, and adjusting treatment frequency.

This stage recommends a prospective study design, typically a single‐center cohort study, combined with case reports when available [30]. The study should comprehensively document sequential modifications in techniques, indications, and patient characteristics, reflecting an iterative optimization process. If existing clinical practice data, such as case reports, sufficiently support the optimization or standardization of acupuncture, prospective cohort studies may not be required. The primary outcomes of this stage include the development of a detailed and optimized acupuncture protocol. This protocol should specify patient characteristics, technical details of the treatment (i.e., type of acupuncture, acupoint prescription, manipulation techniques, needling depth or angle, treatment frequency, and duration), and preliminary data on efficacy and safety. Additionally, a standardized technical training program for acupuncturists will be established for the next stage of study.

#### 3.3.1 Example of Stage 2

Clinically, acupoint sensitization is determined by the acupuncturist's experience, which is not standardized or quantified. A prospective cohort study was conducted, involving 45 patients with knee osteoarthritis, to measure sensitization based on pain threshold (PT) at 13 acupoints and the Ashi point from 12 testing areas around knee [31]. Trained acupuncturists measured PT with the electronic von Frey detector (2390 series, IITC Life Science). Each point was tested twice at an interval of 2 min. If the difference between the two values was greater than 15 g/N, a third measure was made at this point. The average of two values with the smallest difference was recorded as the final PT of the tested acupoint. The acupoints were ranked by the PT value. The five points with lowest PT were identified as low PT acupoints (corresponding to high pain sensitivity), and the five with the highest PT as high PT acupoints (corresponding to low pain sensitivity).

In addition, a standardized acupuncture regimen was developed, including number and selection of needles, manipulation of needles (i.e., needle insertion, depth of needles, angle of needles, lifting and thrusting, twirling and rotating, number of manipulations, needle withdrawal), achievement of Deqi sensation, needle retention time, treatment frequency and duration.

### 3.4 Stage 3 (Exploration): Exploring the Feasibility of Acupuncture Regimen for Conducting a Definitive Randomized Controlled Trial (RCT)

In this stage, researchers would conduct a pilot trial to test the feasibility of the optimized acupuncture intervention and study protocol. This may involve testing in a small group of participants at multiple centers to identify any issues that need addressing before conducting a larger trial. These include establishing rigorous patient inclusion/exclusion criteria, developing targeted recruitment strategies, validating data collection methodologies, standardizing implementation protocols for control group, optimizing training programs for consistent delivery of interventions by acupuncturists, monitoring adherence to treatment protocols, and designing outcome measures that capture the unique effects of acupuncture [32]. Additionally, a critical component of this stage involves addressing variability introduced by acupuncturist expertise, clinical experience, and patient‐practitioner communication dynamics. Researchers should identify and measure variables related to acupuncturist training, such as qualification, experience level, and learning curve, as well as patient responses during treatment. Data analysis should assess the influence of these differences on outcomes, thereby formalizing a replicable acupuncture operational framework that integrates technical standards with adaptive communication strategies.

When the purpose is to investigate an existing treatment or adjust a specific aspect of the existing acupuncture regime, such as determining whether increasing the frequency of acupuncture sessions for treating a certain condition from one to three times per week will result in improved outcomes, stage 2 may be skipped and proceed directly to a feasibility study in stage 3 [33]. It is worth noting that conducting RCTs with sham acupuncture as placebo control may be challenging due to the potential therapeutic effects of sham techniques, which involve non‐penetrative needling or superficial insertion at non‐acupoints [9]. Creating an inert sham acupuncture is difficult, but researchers can consider testing its effectiveness in stage 3 study to determine if it adequately simulates non‐specific effects associated with acupuncture therapy. This will assist in determining if indeed it is an appropriate sham treatment, and play an important role in sample size calculations.

#### 3.4.1 Example of Stage 3

A pilot RCT was conducted to compare the effects of acupuncture at low PT acupoints (corresponding to high pain sensitivity) with acupuncture at high PT acupoints (corresponding to low pain sensitivity) for knee osteoarthritis, aiming to explore the consistency of acupuncture regimen among different operators and the feasibility of the study [34]. Four acupuncturists with at least 3 years of clinical experience were trained to measure PT and select acupoints for intervention. They successfully qualified by selecting the same treatment acupoints for a given patient. Subsequently, they underwent training in the standardized acupuncture protocol and were required to demonstrate proficiency in its implementation, as assessed by an experienced senior acupuncturist. Finally, a dedicated quality controller evaluated their performance of the standardized acupuncture protocol during the study. This trial presented preliminary data on the feasibility of conducting a large trial to test the effects of acupuncture at highly pain‐sensitized acupoints in patients with knee osteoarthritis.

### 3.5 Stage 4 (Assessment): Confirming the Effects by Comparing Acupuncture With Standard Therapy or Sham Acupuncture

When stage 3 has provided evidence that an acupuncture treatment or an optimized acupuncture regimen is feasible, researchers should conduct a definitive RCT to evaluate its effect by comparing acupuncture with standard therapy, sham acupuncture or no treatment. Stage 4 evaluation preferably involves a number of patients and acupuncturists at multiple centers [35]. The trial would be designed according to the principles of the IDEAL framework, with clear reporting of the patients, intervention, control, outcomes, and adverse events.

Due to the complex characteristics of acupuncture, quality control is essential for conducting acupuncture RCT. To ensure reliable and valid results, it is recommended that researchers develop standardized procedures of acupuncture operations based on stage 3 studies and conduct unified training for acupuncturists. Furthermore, establishing supervision mechanisms throughout the research process is crucial. This may involve regular monitoring and evaluation by experienced supervisors with expertise in both acupuncture practice and clinical trial methodology to maintain high‐quality standards.

#### 3.5.1 Example of Stage 4

A three‐arm RCT was conducted to evaluate the effect of acupuncture at highly pain‐sensitized acupoints on knee osteoarthritis [36]. The study included an acupuncture group targeting acupoints with low PT (corresponding to high pain sensitivity), an acupuncture group targeting acupoints with high PT (corresponding to low pain sensitivity), and a waiting‐list group. A total of 666 participants and 10 acupuncturists from four centers were recruited for this study. All acupuncturists underwent standardized training based on the methods used in the feasibility study. Additionally, all researchers received comprehensive training on the Standard Operating Procedure and Case Report Form of the study. Quality controllers regularly visited each center to ensure proper implementation of patient recruitment, random allocation, PT measurement, acupuncture intervention, outcome measurement, and other aspects of the research project. Ultimately, it was found that acupuncture at highly pain‐sensitized acupoints effectively treated knee osteoarthritis.

### 3.6 Stage 5 (Long‐Term Monitoring): Examining the Effects of Acupuncture Treatment Regimen in Clinical Practice

Stage 5 focuses on the long‐term monitoring of acupuncture treatment regimen in routine practice. Given that acupuncture treatments often involve multiple sessions over extended periods, particularly for chronic conditions such as neurological, musculoskeletal, and gastrointestinal disorders, it is essential to evaluate not only short‐term outcomes (as done in IDEAL stages 1–4) but also the sustainability of therapeutic effects and long‐term safety. Considering factors such as disease chronicity, recurrence rates, the complexity of acupuncture protocols, and potential delayed impacts, researchers would conduct comprehensive long‐term follow‐up studies. These studies aim to assess the durability of clinical benefits, refine treatment regimens for optimal long‐term application, and monitor any adverse events that may emerge over time. For instance, in managing chronic insomnia, after an initial intensive treatment phase, ongoing long‐term follow‐up is essential to ensure that improvements in sleep quality are maintained. Follow‐up assessments may be conducted every few months over a period of one to two years to monitor whether patients continue to experience enhanced sleep quality without relapse and whether the frequency of acupuncture treatments can be gradually reduced over time.

Pragmatic RCT is valuable for assessing the long‐term effects of acupuncture in real‐world clinical settings [37]. It can assist in determining whether the positive effects observed during short‐term assessments are sustained over time, as well as identifying any delayed benefits or potential adverse effects. The design of pragmatic RCT should reflect the diagnostic and therapeutic characteristics of acupuncture, such as acupuncture operational details and acupuncture diagnostic and therapeutic thinking. Additionally, a well‐designed registry study, which is a clinical measure of real‐world treatment data, would provide an excellent platform for conducting long‐term evaluations on acupuncture. Since the International Acupuncture Case Registry data collection system's establishment in 2017, extensive data on diagnosis and treatment of acupuncture, as well as its effectiveness evaluation, has been collected from 3404 patients, allowing researchers to integrate and apply clinically meaningful indicators to evaluate real‐world acupuncture effectiveness and safety and explore factors affecting acupuncture effectiveness [38].

#### 3.6.1 Example of Stage 5

We plan to perform a pragmatic RCT to confirm the long‐term effectiveness of acupuncture treatments targeting highly pain‐sensitized acupoints for knee osteoarthritis in a practical clinical setting.

## 4 Discussion

The proposed IDEAL‐Acu framework was specifically designed for evaluating acupuncture interventions, emphasizing the importance of conducting studies at each stage, from proposal to long‐term monitoring of acupuncture regimens. This framework was derived from the IDEAL developed for surgery, with necessary modifications to better accommodate the unique characteristics of acupuncture. For instance, the stage 0 (preclinical studies) may not be essential for every acupuncture regimen unless novel techniques are introduced. Instead, ancient literature and traditional Chinese medicine theories can serve as valuable resources to propose evidence‐based regimens in stage 1, in preparation for stage 2.

Unlike conventional guidelines focused on reporting standards (e.g., CONSORT) [39, 40], the IDEAL‐Acu framework provides a structured, stage‐based approach to guide the entire lifecycle of acupuncture intervention proposal, optimization, exploration, evaluation and longer‐term monitoring. Each stage is designed to address specific research needs and challenges, ensuring both methodological rigor and practical applicability. Stage 1 facilitates the proposal of acupuncture regimens, such as novel acupoint prescriptions or exploration of new disease areas. By integrating evidence from ancient literature and clinical practice, researchers can develop innovative yet evidence‐based interventions for acupuncture. Stage 2 focuses on optimizing or standardizing of proposed acupuncture regimen. For example, iterative refinement of needling techniques, treatment frequency, and practitioner training protocols ensures that the intervention is replicable and potentially effective. Stage 3 tests the feasibility of the optimized acupuncture intervention and study protocol, such as patient recruitment, adherence, and outcome measurement strategies. Stage 4 involves comparing the effects of acupuncture treatments with standard or sham treatments. Although sham acupuncture poses methodological challenges due to its potential regimen effects, careful design and validation of inert sham techniques can enhance the validity of RCTs. Stage 5 evaluates the sustainability of treatment effects and monitors long‐term safety. The focus on real‐world applicability and long‐term monitoring can improve the generalizability of study findings, making them more relevant to clinical practice.

This study has several limitations. First, although extensive interactive discussions were conducted with internal and external experts during the development of the IDEAL‐Acu framework, potential limitations may still exist because the sample of experts might not fully represent all regions and acupuncture traditions. Second, while the IDEAL‐Acu framework is theoretically robust and provides clear recommendations for each stage along with specific examples, its clinical application value and efficacy need to be evaluated with more empirical evidence. Future studies are needed to assess how effectively the framework guides real‐world acupuncture research, improves evidence quality, and influences clinical practice. Third, within the framework, we made sham acupuncture an optional control in Stage 4. Due to the methodological challenges of designing inert sham acupuncture, such as non‐penetrative needling, superficial insertion at acupoints, or par‐acupoint insertion, we did not offer explicit design guidance. Instead, we recommended testing sham acupuncture in stage 3 to determine whether it adequately simulates the non‐specific effects associated with acupuncture treatment.

Overall, the IDEAL‐Acu framework offers a comprehensive, transparent, and reliable approach to evaluating the effects of acupuncture. It complements existing reporting guidelines by providing a structured methodology for the entire lifecycle of intervention proposal, optimization, exploration, evaluation and longer‐term monitoring. By adhering to IDEAL‐Acu, researchers and practitioners can enhance the quality of acupuncture research, inform evidence‐based healthcare decisions, and ultimately contribute to the development and improvement of acupuncture therapies.

## Conflicts of Interest

The authors declare no conflicts of interest.
